# Supplementary material for: Mapping the planet’s critical natural assets
Source: Nat Ecol Evol. 2022 Nov 28;7(1):51–61. doi: 10.1038/s41559-022-01934-5 (PMC9834042; doi:10.1038/s41559-022-01934-5)
Supplement: Supplementary file 2 — Reporting Summary [file 41559_2022_1934_MOESM2_ESM.pdf]

nature portfolio

Corresponding author(s): Chaplin-Kramer

Last updated by author(s): Oct 3, 2022

Reporting Summary

Nature Portfolio wishes to improve the reproducibility of the work that we publish. This form provides structure for consistency and transparency in reporting. For further information on Nature Portfolio policies, see our Editorial Policies and the Editorial Policy Checklist.

Statistics

For all statistical analyses, confirm that the following items are present in the figure legend, table legend, main text, or Methods section.

n/a	Confirmed	
☒	☐	The exact sample size (*n*) for each experimental group/condition, given as a discrete number and unit of measurement
☒	☐	A statement on whether measurements were taken from distinct samples or whether the same sample was measured repeatedly
☒	☐	The statistical test(s) used AND whether they are one- or two-sided<br>*Only common tests should be described solely by name; describe more complex techniques in the Methods section.*
☒	☐	A description of all covariates tested
☒	☐	A description of any assumptions or corrections, such as tests of normality and adjustment for multiple comparisons
☒	☐	A full description of the statistical parameters including central tendency (e.g. means) or other basic estimates (e.g. regression coefficient) AND variation (e.g. standard deviation) or associated estimates of uncertainty (e.g. confidence intervals)
☒	☐	For null hypothesis testing, the test statistic (e.g. *F*, *t*, *r*) with confidence intervals, effect sizes, degrees of freedom and *P* value noted<br>*Give P values as exact values whenever suitable.*
☒	☐	For Bayesian analysis, information on the choice of priors and Markov chain Monte Carlo settings
☒	☐	For hierarchical and complex designs, identification of the appropriate level for tests and full reporting of outcomes
☒	☐	Estimates of effect sizes (e.g. Cohen's *d*, Pearson's *r*), indicating how they were calculated

Our web collection on statistics for biologists contains articles on many of the points above.

Software and code

Policy information about availability of computer code

Data collection	We used InVEST version 3.8 (free and open-source, available at naturalcapitalproject.stanford.edu/software/invest) and Co$tingNature version 3 (free for non-commercial uses, available at www.policysupport.org/costingnature), along with several open-source pre-published datasets.
Data analysis	We used prioritizr available at https://prioritizr.net/

For manuscripts utilizing custom algorithms or software that are central to the research but not yet described in published literature, software must be made available to editors and reviewers. We strongly encourage code deposition in a community repository (e.g. GitHub). See the Nature Portfolio guidelines for submitting code & software for further information.

Data

Policy information about availability of data

All manuscripts must include a data availability statement. This statement should provide the following information, where applicable:

- Accession codes, unique identifiers, or web links for publicly available datasets
- A description of any restrictions on data availability
- For clinical datasets or third party data, please ensure that the statement adheres to our policy

All data and code to run it are available at: https://osf.io/r5xz7/?view_only=d611a688525f4ceb8db4ef4e7528b0e8

Field-specific reporting

Please select the one below that is the best fit for your research. If you are not sure, read the appropriate sections before making your selection.

☐ Life sciences ☐ Behavioural & social sciences ☒ Ecological, evolutionary & environmental sciences

For a reference copy of the document with all sections, see nature.com/documents/nr-reporting-summary-flat.pdf

Ecological, evolutionary & environmental sciences study design

All studies must disclose on these points even when the disclosure is negative.

Study description	We ran spatially explicit models of nature's contributions to people and integrated their outputs along with pre-existing datasets into a multi-objective optimization.
Research sample	Spatially-explicit GIS data
Sampling strategy	Data were resampled from 300 m resolution to 2 km resolution to include in the spatial optimization; otherwise no sub-sampling was performed.
Data collection	Data were provided by coauthors Chaplin-Kramer, Sharp, Mulligan, McIntyre, Spalding, Watson, Keys, and Roehrdanz. Methods to produce each spatial dataset are detailed in the Supplementary Information
Timing and spatial scale	Data are mostly from 2015, but span the 2000-2017 window (annual/snapshot), and are global in extent.
Data exclusions	No data were excluded
Reproducibility	Code is published to allow reproducibility
Randomization	Randomization is not necessary because all data are included in the optimization
Blinding	Blinding is not relevant because all data are included in the optimization

Did the study involve field work? ☐ Yes ☒ No

Reporting for specific materials, systems and methods

We require information from authors about some types of materials, experimental systems and methods used in many studies. Here, indicate whether each material, system or method listed is relevant to your study. If you are not sure if a list item applies to your research, read the appropriate section before selecting a response.

Materials & experimental systems

n/a	Involved in the study	
☒	☐	Antibodies
☒	☐	Eukaryotic cell lines
☒	☐	Palaeontology and archaeology
☒	☐	Animals and other organisms
☒	☐	Human research participants
☒	☐	Clinical data
☒	☐	Dual use research of concern

Methods

n/a	Involved in the study	
☒	☐	ChIP-seq
☒	☐	Flow cytometry
☒	☐	MRI-based neuroimaging
